# Livestock Issues: On Hens and Needles

**DOI:** 10.1289/ehp.113-a370

**Published:** 2005-06

**Authors:** Cynthia Washam

Asian governments alarmed at the unprecedented spread of the deadly H5N1 avian influenza virus are seeking relief in a controversial vaccination program. The Thai government announced in February 2005 that it would join China and Indonesia in vaccinating select healthy ducks and chickens. Vietnam also is considering a vaccination program.

Vaccinations can lessen the risk of influenza by reducing the birds’ chances of infection and minimizing the amount of virus shed through nasal secretions and feces by those that do become infected. But vaccinated chickens can still become infected while showing no symptoms of disease (chickens that have not been vaccinated typically die within 48 hours of infection). For that reason, many countries—including Japan, one of Thailand’s biggest poultry markets—ban imports of vaccinated chickens. Countries therefore usually vaccinate poultry against influenza only as a last resort.

“The concern is that if a vaccine is used, it will be harder to identify the virus,” says epidemiologist Mark Katz of the Centers for Disease Control and Prevention, “and it’s not a guarantee that vaccination will completely eliminate the shedding of virus.”

Asian farmers, though, are running out of options. Mass culling has done little to stem the epidemic. More than 120 million chickens in Vietnam, Thailand, and China died or were destroyed during a three-month period early in 2004. A 2 September 2004 article in *Nature* says many Thai farmers are turning to ineffective black-market vaccines to avoid killing their birds. But black-market vaccines can contain viruses that have not been properly inactivated, and may spur the evolution of even more dangerous strains.

Moreover, the virus poses the serious threat of sparking a worldwide human pandemic. H5N1 is highly virulent in humans, with a death rate of more than 60%. What’s kept the virus in check among humans so far is its inability to spread readily from person to person. Fewer than 10 of the 79 confirmed human cases are thought to have resulted for person-to-person contact—most victims handled infected poultry. Scientists believe, though, that H5N1 could mutate into a strain that spreads as easily among humans as the common cold.

“If you put less virus back into the environment, there’s less chance of transmission,” says David Swayne, laboratory director at the U.S. Department of Agriculture’s Southeast Poultry Research Laboratory. “The negatives of vaccination are small if it’s used properly.”

The best prospects for containing avian flu come from using vaccines in conjunction with rigorous surveillance, quarantines, escape-proof poultry coops, and disinfection of poultry handlers and their equipment. Through much of Southeast Asia, though, low budgets and a weak infrastructure hinder such commonsense measures. Millions of peasants, each raising a dozen chickens in their backyard, are simply beyond the reach of government efforts.

Yet another barrier to stemming the epidemic is the reluctance that developing countries have to reporting news that could hurt their economies. Mainland China, where scientists believe the virus first emerged before 1997, acknowledged avian flu for the first time only in 2004, after outbreaks were reported in several neighboring countries. Chinese scholars later admitted in an article published 16 February 2004 in *Newsweek International* that the virus was rampant in several provinces as early as 2001.

H5N1 has become so entrenched in some regions of Southeast Asia that it has now established a permanent ecological niche in poultry, according to a January 2005 World Health Organization report, *Avian Influenza: Assessing the Pandemic Threat*. “The chance of complete eradication in the near future is very unlikely,” Swayne says.

Still, Swayne sees reason for hope. In a 7 March 2005 review he wrote for the International Society for Infectious Disease, he deemed the commonly used inactivated AI vaccine effective, along with two new vaccines developed for use in Chinese poultry. And government-sponsored vaccination programs such as those in China and Thailand reduce the risk of farmers using black-market vaccines.

Juan Lubroth, a veterinarian specializing in infectious diseases with the Food and Agriculture Organization of the United Nations, agrees with Swayne that progress is being made in the fight against H5N1, albeit slowly. “I think we’ll have a few years to deal with this virus,” Lubroth says. “But during that time, I think we’ll strengthen the veterinary structure in Asia. Ultimately, it will be good for the production of other livestock.”

## Figures and Tables

**Figure:**
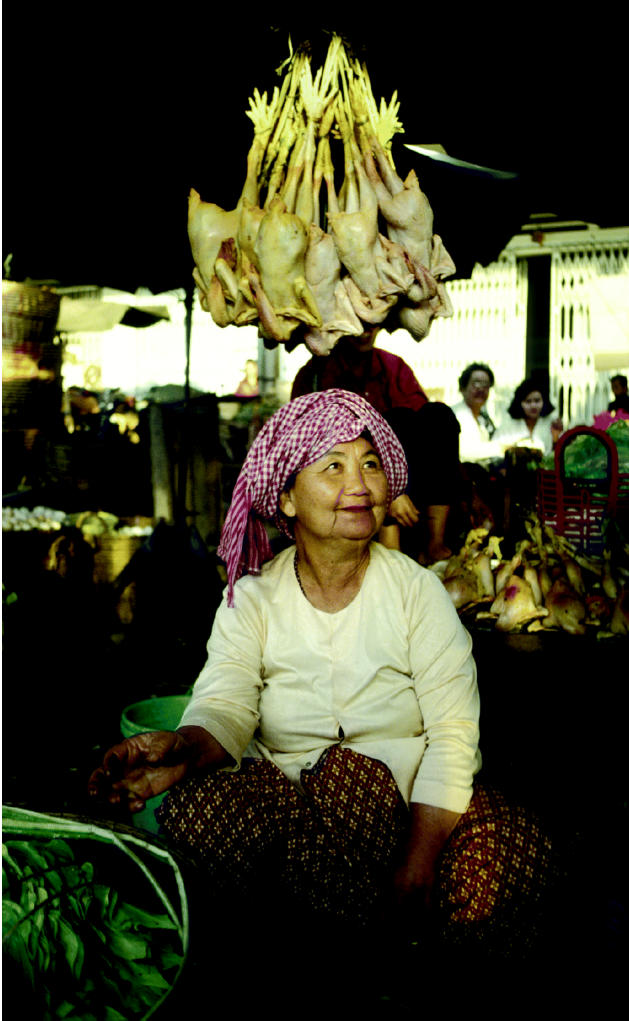
**Preventive medicine?** Several Asian governments have begun vaccinating healthy poultry in hopes of averting the spread of avian flu, but some scientists have concerns about the effectiveness of such programs.

